# Supplementary material for: Shopping festival atmospherics of China’s singles day shopping festival and participants’ perception: Scale development and validation
Source: PLoS One. 2025 Jun 20;20(6):e0324989. doi: 10.1371/journal.pone.0324989 (PMC12180630; doi:10.1371/journal.pone.0324989)
Supplement: Attachment — Submitted filename: Response to Reviewers.docx [file pone.0324989.s003.docx]

# Response to Editor

Dear Editor,

Thank you very much for giving us an opportunity to revise our manuscript. We appreciate the editor and reviewers sincerely for their constructive comments and suggestions to our manuscript entitled “Shopping festival atmospherics of China’s Singles Day Shopping Festival and participants’ perception: Scale development and validation” (PONE-D-24-00459)

Those comments are all· valuable and very helpful for revising and improving our paper, as well· as the important guiding significance to our following research. We have presented detailed response to reviewers’ comments. According to the reviewers’ comments, we have made a careful revision on the original manuscript. All revise contents are marked in yellow in the revised manuscript.

The comments and responses are listed in next pages.

Journal Requirements:

When submitting your revision, we need you to address these additional requirements.

1. Please ensure that your manuscript meets PLOS ONE's style requirements, including those for file naming. The PLOS ONE style templates can be found at

https://journals.plos.org/plosone/s/file?id=wjVg/PLOSOne_formatting_sample_main_body.pdf and

<https://journals.plos.org/plosone/s/file?id=ba62/PLOSOne_formatting_sample_title_authors_affiliations.pdf>

***Response 1:*** Thank you very much for your kind reminder and the paper submission template.

We have carefully revised the paper to meet PLOS ONE's style requirements.

2. You indicated that ethical approval was not necessary for your study. We understand that the framework for ethical oversight requirements for studies of this type may differ depending on the setting and we would appreciate some further clarification regarding your research. Could you please provide further details on why your study is exempt from the need for approval and confirmation from your institutional review board or research ethics committee (e.g., in the form of a letter or email correspondence) that ethics review was not necessary for this study? Please include a copy of the correspondence as an "Other" file.

***Response 2:*** Thank you for your kind reminder. We will explain it from the following aspects:

Firstly, this study attempts to extend the dimensions and attributes of ambiance in online promotions. Secondly, the sample data of the study is only about the consumption behavior and intention of the online shopping festival, and does not involve the data of the medical field.

Finally, in order to meet the ethical norms of the journal and ensure the scientific nature of the research, the Scientific Research Office of "Yiwu Industrial and Commercial College" issued an "Ethics Committee review approval". I hope I can get your approval. See Annex 1 for details.

3. Please provide additional details regarding participant consent. In the ethics statement in the Methods and online submission information, please ensure that you have specified (1) whether consent was informed and (2) what type you obtained (for instance, written or verbal, and if verbal, how it was documented and witnessed). If your study included minors, state whether you obtained consent from parents or guardians. If the need for consent was waived by the ethics committee, please include this information.

***Response 3:*** Thank you very much for your attention and reminder of the research sample. We have screened the questionnaire data one by one and made the following adjustments.

(1) First of all, the survey is voluntary. Secondly, before filling in the questionnaire, the respondents were given instructions to fill in the questionnaire, including the purpose and main content of the questionnaire. The respondents could decide whether to participate in the social survey or not. Finally, the privacy protection of participants is explained.

(2) We designed the questionnaire and entrusted it to the largest questionnaire service company in China—wjx.cn (<https://www.wjx.cn/>) for questionnaire collection. Details are as follows:

1) The authors designed the questionnaire according to the purpose of the study.

2) Using the service function of the wjx.cn (https://www.wjx.cn/), the questionnaire can be directly read and filled out on the mobile phone.

3）Entrust wjx.cn (https://www.wjx.cn/) (the largest questionnaire service platform in China) to sign the service agreement to determine the demographic attributes of the questionnaire collection, the number of questionnaires and the reward of the questionnaire.

4）According to the reading of the survey instructions, the respondents decide whether to fill in the questionnaire voluntarily. They can scan the QR code or open the link to fill in the questionnaire through their mobile phones, and receive the reward after completing the questionnaire.

5）Finally, the collected questionnaires were preprocessed, and the unreasonable questionnaires such as incomplete questionnaires and extreme answers were eliminated. The final valid questionnaires were used for data statistical analysis.

(3) According to the feedback of the wjx.cn (https://www.wjx.cn/), after answering each valid questionnaire, the respondents will receive a certain red envelope reward. At the same time, the IP address and filling time of each questionnaire can be traced to the source, so as to ensure the authenticity and validity of the questionnaire.

(4) A total of 6 minors participated in this questionnaire survey. In order to avoid the ethical issues involved in the survey data of minors, after careful consideration, we decided to eliminate the data involving six minors and then conduct the data analysis again.

(5) Six questionnaires from minors were removed from the study sample data, leaving 686 valid questionnaires.

(6) The following are the instructions for filling out the questionnaire:

Dear respondents：

Hello! In recent years, with the upgrading of online shopping consumption demand, the online shopping festival with the "Double 11 online shopping Carnival" has shown great consumption potential. As college teachers, we conducted a social survey on consumers' perception of "shopping festival atmosphere" in the "Double 11 Online Shopping Carnival". We hope to form an effective scale for "shopping holiday atmosphere" through statistical analysis of the collected questionnaire data. The data obtained from this questionnaire is only used for academic research. It will not disclose your personal privacy and personal views, and will not be used for any commercial purposes. Please feel free to fill in the questionnaire. Finally, in order to thank you for your support for this social survey, after filling out the questionnaire, there will be a red envelope reward.

4. Thank you for stating the following financial disclosure:

"The Philosophy and Social Science Planning Project of Zhejiang Province, No. 23NDJC414YBM"

Please state what role the funders took in the study. If the funders had no role, please state: "The funders had no role in study design, data collection and analysis, decision to publish, or preparation of the manuscript."

If this statement is not correct you must amend it as needed.

Please include this amended Role of Funder statement in your cover letter; we will change the online submission form on your behalf.

***Response 4:*** Thank you very much for your kind reminder to us on this issue, for which we make the following explanation.

It should be noted that, on the one hand, the head of the funded project is Professor Shao Chunling. On the other hand, the first author of this paper is a core member of this funded project.

The funder, Professor Chunling Shao, did not play a role in the study design, data collection and analysis, decision to publish, or preparation of the manuscript. However, in the process of paper revision, Professor Shao Chunling supervised and funded the paper layout fee. Therefore, the authors of this paper unanimously agreed to add Professor Shao Chunling to the author sequence of this paper.

5. We note that your Data Availability Statement is currently as follows: All relevant data are within the manuscript and its Supporting Information files.

Please confirm at this time whether or not your submission contains all raw data required to replicate the results of your study. Authors must share the “minimal data set” for their submission. PLOS defines the minimal data set to consist of the data required to replicate all study findings reported in the article, as well as related metadata and methods (https://journals.plos.org/plosone/s/data-availability#loc-minimal-data-set-definition).

For example, authors should submit the following data:

- The values behind the means, standard deviations and other measures reported;

- The values used to build graphs;

- The points extracted from images for analysis.

Authors do not need to submit their entire data set if only a portion of the data was used in the reported study.

If your submission does not contain these data, please either upload them as Supporting Information files or deposit them to a stable, public repository and provide us with the relevant URLs, DOIs, or accession numbers. For a list of recommended repositories, please see

https://journals.plos.org/plosone/s/recommended-repositories.

If there are ethical or legal restrictions on sharing a de-identified data set, please explain them in detail (e.g., data contain potentially sensitive information, data are owned by a third-party organization, etc.) and who has imposed them (e.g., an ethics committee). Please also provide contact information for a data access committee, ethics committee, or other institutional body to which data requests may be sent. If data are owned by a third party, please indicate how others may request data access.

***Response 5:*** Thank you very much for your suggestions on the data analysis part. We have also made the following adjustments.

Firstly, we will upload the data set used in this study as required.

Second, detailed data reports (such as mean and standard deviation) have been added to this study in section 3.2 (page 13) Table4.

Finally, our attempts to submit the data in the data repository were always unsuccessful, so we submitted a data link (url: <https://pan.quark.cn/s/3dce718cd231> ), through which we could directly access the questionnaire data used in this study.

6. Please include captions for your Supporting Information files at the end of your manuscript, and update any in-text citations to match accordingly. Please see our Supporting Information guidelines for more information: http://journals.plos.org/plosone/s/supporting-information.

***Response 6:*** Thank you very much for your many suggestions during this submission process. We must carefully revise the paper according to the requirements of the editors and reviewers, and upload the repaired paper within the prescribed time.

# Response to Reviewer 1 Comments

**1. Summary**

Thank you very much for taking the time to review this manuscript. These opinions help to improve the academic rigor of our article. Please find the detailed responses below and the corresponding revisions highlighted in the re-submitted files.

**Reviewer #1:** The paper discusses atmosphere scale development based on the successful online shopping SDSF event. The topic is relevant and the methodological approach is basically adequate. However, I have some major concerns about the presentation in the paper and the underlying data collection and analysis:

1. The literature relies mainly on elder articles. Many current and relevant publications are not reflected. So, e.g., Cheng and Chen (2023) discuss an atmosphere scale development in a detailed and methodologically advanced manner. Also, the author cites many times DeVellis (2003) where as DeVellis and Thorpe (2021), the advanced and extended fifth edition of this standard book provides advanced insights. Also "Kolter 1973" is cited, but the author wants to cite Kotler 1973, the famous atmosphere article by the famous marketing professor (:O). Few scientific articles cited in the paper are from 2015 onwards (exactly: 10), the paper needs a complete refresh of its references and consequently a discussion of the state of the art based on this refreshing.

**Response 1:** I am very glad that you have provided me with two important references, which have provided great help for the revision of this paper.

Firstly, we carefully listen to your suggestions and update the literature cited in the paper in a timely manner by reading the relevant literature in the past 5 years.

Secondly, according to the relevant literature, theoretical basis and research methods of the research topic in this paper, we have made a lot of changes in the introduction, literature review and discussion of research results.

Finally, Thank you again for the paper (Cheng and Chen, 2023) and the latest reference book (DeVellis and Thorpe, 2021). The specific changes are as follows.

（1）In the introduction, first of all, the previous content and paragraph were adjusted. Secondly, the research literature on "China's Singles Day Shopping Festival" and "festival atmosphere" in the past five years is focused on. Finally, a description of the value of the research is added.

**Page1 line 40-50**

Improving the variety of promotion categories and the interest of promotion activities are very effective promotion strategies during online shopping festival [9]. Information quality, product quality and cost savings can significantly improve consumer satisfaction with online shopping festivals [10]. Later, researchers found that the Double 11 online shopping festival was not only a promotional activity, but also an entertainment for participants. Collaboration between Chinese e-commerce platforms and merchants in terms of product promotion and atmospheric marketing strategies is key to the success of the SDSFs [9]. Xu, Wang, and Zhao (2020) posit that SDSFs creates unique shopping scenarios that can more effectively stimulate consumers' emotions and influence their shopping behaviors [11]. Hedonistic shopping has become the main motivation for consumers to participate in online shopping festivals, in addition to saving money [12].

**Page2-3 line71-99**

The influence of shopping festival atmospheres on consumer behavior in SDSFs has also been the subject of research by scholars in the field. In a study conducted by Yang, Li, and Zhang (2018), it was discovered that the atmosphere surrounding SDSFs was negatively correlated with consumers' intentions to engage in sustainable consumption [23]. This atmosphere has become SDSFs most important marketing tool for stimulating consumer purchases [24]. The shopping festival atmosphere plays a positive moderating role between promotional stimulation and satisfaction, as well as between satisfaction and the intention to continue participating [25]. Despite the considerable attention that research on the atmosphere of online shopping festivals has attracted, there is still no consensus on the exact connotations of this phenomenon. There are no clear conclusions about the difference between the atmosphere of online shopping festivals and that of traditional shopping environments, and there is a lack of scientifically valid scales to describe this unique atmosphere.

"SDSFs" created by Alibaba is the earliest online shopping festival in China (2009.11.11), with the highest popularity, the most extensive consumer participation and the most influential [8, 10]. The success of other online shopping festivals (e.g. JD 6.18) also draws lessons from the successful experience of SDSFs. The rapidly growing sales data of "SDSFs" over the years and the popular discussion on social platform [1, 23]. SDSFs has undergone a significant transformation, evolving from a focus on simple price promotions to the development of comprehensive marketing campaigns that prioritize the creation of an entertaining and interactive atmosphere [26]. Given the relatively standardized and homogeneous nature of e-commerce activities worldwide, the findings of the survey on Chinese e-commerce platforms can be extrapolated to a number of other platforms globally. This offers valuable insights and recommendations for other countries preparing for similar shopping festivals. Therefore, the "SDSFs" is not only a typical representative of online shopping festival, but also provides reference value for the development of similar online shopping festivals.

This study posit that atmospherics represents one of the most salient characteristics of large-scale online sales events. In accordance with the nomenclature of the SDSFs, the present study endeavors to encompass the comprehensive dimensions of "shopping festival atmospherics" (SFA).

（2）On the one hand, the "S-O-R theory" is added as a theoretical basis in the first part of the literature review. On the other hand, in the "atmosphere definition", "physical store atmosphere", "online store atmosphere", "cultural atmosphere", "festival atmosphere" sections, the relevant descriptions have been revised and the literature has been updated in the past five years.

**Page 3-4 line111-142**

2.1	Theoretical foundation

The S-O-R (Stimulus-Organism-Response) theory is derived from the S-R (Stimulus-Response) theory of behavioral psychology [27]. The S-O-R model was first proposed by Woodworth, and he explained that stimuli elicit different responses depending on the state of the organism[28]. Environmental psychologists use the S-O-R paradigm to describe the external stimulus, intervention variables, and behavioral outcomes [29]. The salient feature of the S-O-R theory is that consumer decision making goes through three distinct stages, i.e. external stimuli (S), affecting individual emotional states (O), and finally triggering behavioral responses (R)[30]. S-O-R theory is widely used to study offline and online consumer psychology and behavior. Currently, popular online consumption scenarios include: Fashion e-commerce retail [31], TikTok store [32], Live streaming e-commerce [33].

Kotler (1973) defined atmosphere as an environment designed to elicit emotional and behavioral responses from customers [19]. In 1974, Mehrabian and Russell pioneered the use of the S-O-R paradigm to examine the influence of atmospheric factors on consumer behavior [29]. In the positivist paradigm (S-O-R), numerous scholars argue that atmosphere is typically external stimulus (S) that influences the consumers' internal organism (O), subsequently leads to behavioral response (R) [34-36]. In recent years, the S-O-R framework has been employed extensively to examine the online shopping atmosphere in a multitude of contexts. For example, the visual complexity of shopping websites [37] and the website informativeness and effectiveness of information content [38] have been identified as key factors. Chen and Li (2019) posited that the atmosphere of shopping festivities is the primary factor influencing consumer purchasing intentions [24]. Xie et al. (2023) investigated the influence of the atmosphere of an online shopping festival as a stimulus factor on consumers' intention to engage in continued participation [26]. In accordance with the S-O-R theory, Cheng and Chen (2023) devised a creative atmosphere scale, and its process and methodology provide a very valuable reference for this research [39]. This study puts forth the concept of shopping festival atmosphere (SFA) from the vantage point of consumption perception stimulus, with a foundation in the S-O-R framework. SFA is evidenced by the implementation of extensive product promotions, the configuration of online platforms, the incorporation of entertainment games, the facilitation of social interaction, and other forms of expression. SFA is a stimulating element (S), consumers experience the joy, stimulation, and excitement associated with shopping carnivals (O), ultimately exhibiting a tendency to engage in continuous participation (R).

**Page 4 line162-164**

2.3 Atmospherics of physical stores

In his seminal work, Kotler (1973) posited that the store atmosphere encompasses four dimensions: visual, auditory, tactile, and olfactory [19].

**Page 5 line172-173**

Similarly, the store atmosphere can be divided into three dimensions: social categories (3 items), physical categories (3 items), and ambient features (2 items) [42].

**Page 5 line189-190**

There have been many interesting findings on the study of store atmosphere on consumer behavior.

**Page 5 line193-200**

In young shoppers' sections and pop-up stores, playing modern and stylish music can increase their desire to stay in the mall [50]. In global apparel retailer's stores, customers in a non-English speaking country are more likely to buy when music is played in English [53]. The olfactory congruence of a shopping mall has a positive effect on bridging the mall atmosphere and brand image [54]. In young shoppers' sections and pop-up stores, playing modern and stylish music can increase their desire to stay in the mall [31]. The visual atmosphere of a coffee shop can have an impact on consumers' expectations of different coffee flavors [55].

2.4 Atmospherics of online stores

**Page 6 line 224-226**

Some scholars differentiate between the various elements that comprise the atmosphere of online stores, including information, navigation quality, as well as platform design [60, 61].

**Page 6 line 236-243**

Ettis (2017) found that the blue-hues background of an online store induces more consumers’flow (Enjoyment and concentration) than yellow[65]. The unique shopping atmosphere of e-commerce live streaming is reflected in three aspects: bullet screen, guide information as well as parasocial interaction [66]. In live streaming e-commerce, participants generate high arousal when they are in an atmosphere with complex background images and fast-paced background music, and fast-paced music seems to better meet the needs of consumers [33].

2.6.1 cultural cues

**Page 7 line 285-286**

Consuming during the “SDSFs”is considered a ritualistic activity [35].

2.6.2 social cues

**Page 8 line 330-335**

The atmosphere of participation in the online shopping festival will affect consumers through the medium of social interaction with the media and with other people [24]. Extensive social interaction online and offline is also a major stimulus for a large number of consumers to participate in the SDSFs [9, 25]. The social interaction atmosphere will stimulate the excitement of customers, thus enhancing the intention of consumers to continue to participate in the SDSFs [26].

2.6.3 physical cues

**Page 9 line244-346**

In order to enhance customer purchasing behavior, numerous retailers dedicate a considerable amount of time and resources to the implementation of distinctive merchandise displays at festivals, with the objective of attracting potential customers [75]. The strong technical and service support provided by Alibaba makes consumers feel the“fairness atmospherics” of the China’s Singles Day Shopping Festivals [25].

2.6.4 online cues

**Page 9 line 356-359**

An aesthetically pleasing online store design and intuitive navigation facilitate consumer engagement and enhance the shopping experience on Singles' Day [35]. It is consistent with the research conclusion of Shafiee and Bazargan (2018) [76] in shopping loyalty.

**Page 9 line 361-363**

The strong technical and service support provided by Alibaba makes consumers feel the“fairness atmospherics” of the China’s Singles Day Shopping Festivals [25].

（3）In materials and methods, according to your suggestion, combined with the research methods of previous literature (Dimitrova, Ilieva, and Stanev, 2022; Cheng and Chen, 2023) , the data analysis was re-carried out.

**Page13 line 453-455**

In order to ensure the scientific and normative nature of the research, the collected data can be randomly divided into two parts. According to the research method and process of Dimitrova, Ilieva, and Stanev (2022), EFA and CFA were performed on the two parts of data respectively [80].

For specific modifications, please see Respond 2.

（4）In the discussion and summary section, the research conclusions are divided into two parts: theoretical significance and practical significance, and compared with the relevant research results in recent years to further enrich the value of this research.

4. Discussion and conclusions

4.1Theoretical implications

**Page18 line 540-554**

This research has a number of theoretical implications. First, according to the S-O-R theory, which focuses on "environmental stimuli can trigger individual emotional responses", combined with previous research findings, a shopping festival atmosphere scale was developed. In recent years, with the widespread attention of scholars on online shopping atmosphere, scholars have described the connotation of shopping festival atmosphere from different perspectives [24, 26]. The research findings of this paper not only establish the concept of shopping festival atmosphere, but also expand its theoretical connotation and application.

Secondly, the SFA scale developed in this paper is different from previous measures of atmosphere because it contains three components: cultural, social, and online atmosphere, and it is described in detail. The shopping festival atmosphere not only adds the concept of "festival" to the original online shopping atmosphere, but also takes into account China's unique cultural background and the most extensive online and offline social interaction. This concept extends the application of atmosphere research to the phenomenon of "online shopping festival". The connotation of the shopping festival atmosphere identified in this study is discussed as follows:

**Page18 line 556-557; line 560-561; line 562-565**

Cultural clues refer to the particularity and sense of ritual that must be consumed in online shopping festivals. Just as Zhao and Wan (2017) found in their study that participating in SDSFs is a ritual event [35]. The Confucian values of "keeping face" and "listening to others" exert a moderating influence on the effect of the atmosphere of the online shopping festival on purchase intention [24]. The happily crowded environment (“Renao” in the Chinese language) is a positive atmospheric cue for Chinese participants.

**Page18-19 line 568-576**

Social cues mean that participants actively participate in the most extensive topic communication and knowledge sharing during the SDSFs, both offline and online. The extensive social interaction on the shopping day (such as topic discussion, shopping communication, experience sharing, etc.) has gradually become the biggest feature of Singles Day shopping day [9, 25]. Through online and offline advertising, single consumers can establish a sense of identity with Singles Day [35]. Furthermore, Chen and Li (2020) demonstrated that the festive atmosphere exerts an influence on consumers, which is shaped by the social medias and the people around them [9]. All of these social environments should ultimately result in a crowded atmosphere that is safe, secure and happy. Otherwise, participants may experience negative emotions and misbehave [15, 18].

**Page19 line 578-587**

Online clue is the evaluation of the comprehensive service of the network platform by the consumers participating in the online shopping festival. As an online sales event, the SDSF must change for the special day. This study found that in addition to the traditional online climate of information, efficiency and entertainment, the fair climate was also a new feature perceived by participants. They need very good deals [25]. They don't want to be cheated by artificially inflated prices and they don't want to go to great lengths to play the coupon game. In addition, the authors found that size itself is an attribute of online atmosphere. Studies by Tzeng, Ert, Jo et al. (2020) show that product information and product quality are prerequisites for consumers to feel satisfied after participating in online shopping festivals [10]. The website informativeness and effectiveness of information content can help to improve consumer satisfaction and purchase intention [38].

**Page19 line 592-605**

4.2 Managerial implications

This study has established a reliable scale for measuring the "shopping festival atmosphere," and has also provided managers with a tool for objectively understanding consumers' perception of the shopping festival atmosphere. The results of this study have significant implications for the practice of management, which can be grouped into many areas: Firstly, managers are able to utilize this scale to ascertain whether the shopping festival atmosphere has been successfully created, particularly when the SFA is low. Subsequently, managers are able to modify their strategies in accordance with the aforementioned findings. Secondly, the utilization of cultural, social and online cues can also serve as a valuable means of evidence for managers seeking to establish a shopping festival atmosphere. Thirdly, these cues can be integrated with actual circumstances to develop innovative designs that enhance consumers' purchase intention during the shopping festival. Ultimately, the findings of this study offer valuable insights that can inform the successful implementation of online shopping festivals in other countries, particularly in light of the growing prevalence of such events in China and the global convergence of e-commerce platforms.

**Page19 line 607-635**

5.Limitations and future research

In summary, the present study develops a measurement scale for “shopping festival atmospherics” and provides several novel atmospheric cues extracted from China’s SDSF. Given the dearth of research in this field, the authors hope this study can provide information useful for future investigations of online sales events.

In summary, this study developed a "shopping festival atmosphere" measurement scale and extracted some novel atmosphere cues from the SDSFs. Given the dearth of research in this field, however, some limitations were also identified that need to be addressed and considered.

Firstly, the questionnaire data in this paper was collected only once. However, recent studies on scale development have recommended that questionnaires be collected at least twice. According to the reasonable practice of scale development in previous literature, this paper randomly divided the collected questionnaires into two parts, and carried out EFA and CFA, respectively to ensure the standardization of scale development.

Secondly, the questionnaire in this study was collected in the form of online response, and a small amount of questionnaire data would become invalid due to random filling by the respondents. At the same time, the questionnaire samples collected in this study were only Chinese, and no foreigners were involved.

Thirdly, regarding the scale development of shopping festival atmosphere, this study focused on the efforts of shopping platforms, consumer behavior and feelings. However, this study ignores the influence of different attributes of goods (such as hedonic and practical) on the atmosphere of the shopping festival, as well as the influence on shopping psychology and behavior.

Some scholars have studied "shopping festival atmosphere" (SFA) and found that this factor has a negative impact on consumption intention and behavior. The scale development of SFA in this study provides a theoretical basis for the future research on the consumption behavior of this factor. In the future, the research on SFA can draw on the scale of this study, and analyze the connotation of online or offline SFA. The online shopping festival has entered the era of live e-commerce, and the "shopping festival atmosphere" under this situation has a new connotation, which needs to be further explored. Many scholars have studied the shopping atmosphere of live e-commerce, which also provides a basis for future research.

**2.** The author refers to Churchill and others for scale development. However, only one survey is conducted in contradiction to Chruchill's two surveys (after eliminating and extending the item batteries). I miss a discussion why this is adequate. Just to mention Cheng and Chen (2023): They use two surveys, and the second one is based on a modified questionnaire. Also, the search for items in the construct development phase during the semi-structured interviews is not convincing: The interviewers focus on the SDSF event concerning atmosphere (positive aspects: "Can you list some atmospheric cues you feel during the SDSF?"). They completely miss the aspects that participants miss during an SDSF event. Again, look at Cheng and Chen (2023) for improvements: They ask what "defines" "good" atmosphere at an event.

**Response 2:** Thank you for pointing out this issue, also, thank you very much for the references.

We have carefully read the literature you provided, which is very inspired, and also provides a good research reference for the modification of this article. Based on our understanding, comment 2 involves two aspects that need to be addressed. The first concern is that “there should be two questionnaires, and the second questionnaire survey is based on modified questionnaires”. Our explanations are as follows:

According to Churchill (1979) and Cheng & Chen (2023), the purpose of the two data collections was to perform an exploratory factor analysis (EFA), and the second was to perform a confirmatory factor analysis (CFA) test. This is indeed a shortcoming of this study. In order to ensure the scientific and normative nature of the research, after reviewing the research literature in recent years, it was found that the collected questionnaires could be randomly divided into two parts. Then exploratory factor analysis (EFA) and confirmatory factor analysis (CFA) were performed (Dimitrova, Ilieva, and Stanev, 2022). Based on this, in order to ensure the scientific and reasonable development process of the scale, this paper decided to re-analyze the data.

Firstly, a total of 686 valid questionnaires were collected (6 questionnaires involving minors had been eliminated) and randomly divided into two groups by using SPSS22.0, with 343 questionnaires in each group. The proportions of gender, age, income, education, occupation, participation counts, and chat counts were maintained in the subsample. The results of the chi-square test of independence indicated that there were no statistically significant differences between the participants in the two subsamples with respect to the selected characteristics. Secondly, the first group used SPSS22.0 to conduct exploratory factor analysis (EFA) test, and optimized the questionnaire items (deleted unreasonable items) according to the test standard of exploratory factor analysis (EFA). Finally, AMOS22.0 was used to conduct confirmatory factor analysis (CFA) test on another group of 343 questionnaires to further optimize the items, and finally an effective scale on "shopping festival atmosphere" was formed. Please refer to Sec. 3 for the specific changes and the latest data analysis results.

The specific modification content and the latest data analysis results are as follows.

**Page 9-10 line 365-374**

3. Materials and methods

The present study adopts Churchill (1979) conventional procedures for developing measures[78]. Following Churchill (1979) eight-step measurement development paradigm, the present study posits that scale development can be divided into three principal stages: (1) initial item generation, (2) purification of the measurement scale, and (3) reanalysis of measures [78]. Additionally, this study draws upon the methodology and research process of Cheng and Chen (2023) in formulating the creative atmosphere scale [39] , as detailed in Table 1. below.

**Table 1. The Process of the SFA Scale Development**

| **Main Processes** | **Detailed Description** | **Implementation Results** |
| --- | --- | --- |
| 1. Initial item  generation | 1. Literature Review | Online shopping festival, atmosphere |
|  | 2. In-depth interview (n = 23) | Consumers participating in the SDSFs |
|  | 3. Content analysis | Produce to 4 categories. |
|  | 4. Content validity (2 experts) | Final results: 43 generated items. |
| 2. Purifying the measurement scale | 1. Refinement of instruments  (panel of experts to obtain content validity)  2. Data collection  3. Purifying the measurement  (Sample 1, n = 343), The EFA factor loadings and the test of reliability (Cronbach's α) were employed to reduce the scale. | Removed "Physical cues";  Removed and optimized 24 items that were not suitable for "SFA";  Final results: 19 items belonging to 3 dimensions: 1) cultural cues (6 items), 2) social cues (6 items), and 3) online cues (7 items). |
| 3. Reanalysis  of measures | 1. Data collection (Sample 2,  n = 343); The CFA was conducted with Sample 2, utilizing a three-dimensional model.  2. Criterion-related validity  3. Construct validity analysis | Reduce one item;  The CFA results confirm the scale's strength, with 18 items:  1) cultural cues (6 items);  2) social cues (6 items);  3) online cues (6 items). |

Note: SDSFs = China’s Singles Day Shopping Festivals; SFA= Shopping festival atmospherics;

EFA = exploratory factor analysis; CFA = confirmatory factor analysis.

**Page 9-11 line 375-408**

3.1	Initial item Generation

Following DeVellis (2021) (DeVellis, 2003)’s recommendation, there are three main steps:

*Literature Review*

*In-depth interviews,*

*Content analysis.*

Two research assistants corrected errors and then checked the machine transcription. Two experts coded the transcriptions, assessing the content validity and relevance of the items. They also further assess and review the applicability of the classifications attributed to each item. This resulted in 43 coarse items after synthesizing all atmospheric cues reported by respondents.

These items have been translated into English and are presented in Table 2.

**Page 12 line 430-451**

3.2 Purifying the measurement scale

According to the survey instructions, the respondents decide whether to fill in the questionnaire voluntarily. They can scan the QR code or open the link to fill in the questionnaire through their mobile phones, and receive the reward after completing the questionnaire. The total sample size is 738. After deleting uncertain samples (e.g., incomplete answers, all the same answers, minors), the valid sample size is 686 (Six samples involving minors were deleted from the original data). Table 3 displays the profile of the respondents. As shown in Table 3, more than 98% of the respondents have participated in SDSFs, while approximately 80% participated in SDSFs almost every time. Approximately 75% of the respondents are from the young generation aged 20 to 40. However, there are still a quarter of the respondents who are middle-aged. In addition, more than half of the respondents have frequent chat experiences (5 to 19) during SDSFs. The demographics show that SDSF is indeed an influential sales event attracting a large participant population.

**Table 3. Descriptive Statistics of Respondents (Sample 1 and Sample 2)**

| **Variable** | | **Sample 1 (n = 343)** | | **Sample 2 (n = 343)** | |
| --- | --- | --- | --- | --- | --- |
| Gender | Male | 157 | 45.77% | 166 | 48.40% |
|  | Female | 185 | 53.94% | 177 | 51.60% |
| Age | 19 to 25 | 80 | 23.32% | 86 | 25.07% |
|  | 26 to 30 | 73 | 21.28% | 75 | 21.87% |
|  | 31 to 40 | 110 | 32.07% | 98 | 28.57% |
|  | 41 to 50 | 65 | 18.95% | 71 | 20.70% |
|  | 51 to 60 | 14 | 4.08% | 12 | 3.50% |
|  | Greater than 61 | 1 | 0.29% | 1 | 0.29% |
| Education level | Middle school | 24 | 7.00% | 20 | 5.83% |
|  | High school | 47 | 13.70% | 47 | 13.70% |
|  | Bachelor’s degree | 219 | 63.85% | 221 | 64.43% |
|  | Graduate degree | 49 | 14.29% | 53 | 15.45% |
|  | Post-graduate degree | 4 | 1.17% | 2 | 0.58% |
| Occupation | Working | 216 | 62.97% | 218 | 63.56% |
|  | Student | 59 | 17.20% | 61 | 17.78% |
|  | Freelancer | 42 | 12.24% | 36 | 10.50% |
|  | Retired | 1 | 0.29% | 1 | 0.29% |
|  | Other | 25 | 7.29% | 27 | 7.87% |
| Monthly income | Less than CNY 1000 | 31 | 9.04% | 41 | 11.95% |
|  | CNY 1001 to 3000 | 39 | 11.37% | 37 | 10.79% |
|  | CNY 3001 to 5000 | 55 | 16.03% | 47 | 13.70% |
|  | CNY 5001 to 8000 | 115 | 33.53% | 111 | 32.36% |
|  | CNY 8001 to 15000 | 66 | 19.24% | 76 | 22.16% |
|  | CNY 15001 to 20000 | 31 | 9.04% | 23 | 6.71% |
|  | Greater than CNY 20001 | 6 | 1.75% | 8 | 2.33% |
| Participation counts | Every time | 168 | 48.98% | 142 | 41.40% |
|  | Almost every time | 113 | 32.94% | 141 | 41.11% |
|  | Several times recently | 58 | 16.91% | 54 | 15.74% |
|  | Never | 4 | 1.17% | 6 | 1.75% |
| Chat counts during SDSFs | Greater than 20 | 22 | 6.41% | 20 | 5.83% |
|  | 10 to 19 | 59 | 17.20% | 55 | 16.03% |
|  | 5 to 9 | 139 | 40.52% | 135 | 39.36% |
|  | 1 to 4 | 116 | 33.82% | 126 | 36.73% |
|  | Never | 7 | 2.04% | 9 | 2.62% |

**Page 13-14 line 453-460**

In order to ensure the scientific and normative nature of the research, the collected data can be randomly divided into two parts. According to the research method and process of Dimitrova, Ilieva, and Stanev (2022), EFA and CFA were performed on the two parts of data respectively [80]. The numerical characteristics (mean and standard deviation) of the survey sample1 and sample2 are shown in Table 4.

**Table 4 The measure of the sample data (n=686)**

|  | **Sample 1 (n = 343)** | | **Sample 2 (n = 343)** | |
| --- | --- | --- | --- | --- |
| **Factor/Item** | **Mean** | **Standard Deviations** | **Mean** | **Standard Deviations** |
| Superstars and celebrities are active that day. | 5.379 | 1.470 | 5.466 | 1.416 |
| Most friends in my WeChat circle participate. | 5.207 | 1.469 | 5.440 | 1.406 |
| I feel the “Renao” (happily crowded) atmosphere. | 5.122 | 1.574 | 5.239 | 1.457 |
| Every year the SDSFs is reported as the hot event. | 5.172 | 1.542 | 5.309 | 1.508 |
| I receive many shopping strategies from Xiaohongshu, WeChat, and other social media. | 5.052 | 1.594 | 5.233 | 1.531 |
| Participants share their funny experiences. | 5.050 | 1.597 | 5.265 | 1.466 |
| I feel the SDSFs is a special day. (16) | 4.606 | 1.754 | 4.633 | 1.832 |
| I have the ritual feeling, so I must buy something. | 4.962 | 1.648 | 4.921 | 1.612 |
| I prepare a long time for the day every year. (18) | 4.799 | 1.718 | 4.831 | 1.790 |
| Shopping on Singles Day is a treatment for loneliness. | 5.012 | 1.656 | 5.149 | 1.790 |
| Buy! Buy! Buy! The consumerist culture is the point. | 5.096 | 1.649 | 5.058 | 1.657 |
| I am pressed for time. I have to wait for the next year if I miss the deal. | 5.073 | 1.589 | 5.082 | 1.750 |
| Most of China’s e-commerce companies take part in the SDSFs. | 5.452 | 1.434 | 5.536 | 1.468 |
| The red background color and red envelopes highlight the joyous and festive atmosphere. | 5.531 | 1.456 | 5.504 | 1.535 |
| The logo, discount tag, and virtual division settings make the SDSFs more informative. | 5.052 | 1.661 | 5.105 | 1.602 |
| A large number of brands, vendors, and items take part in the SDSFs. | 5.003 | 1.563 | 5.047 | 1.616 |
| Many online games increase participants’ pleasure. | 5.216 | 1.593 | 5.210 | 1.570 |
| The layout and process designed for both desktop and mobile shopping make the SDSFs more effective. | 5.239 | 1.599 | 5.359 | 1.575 |
| The sales are fair. | 5.044 | 1.575 | 5.090 | 1.591 |

**Page 14-15 line 462-463; line 470-471; line 478-479;482**

*Purifying the measurement.* The present study employs exploratory factor analysis (EFA) with a varimax rotation to determine the dimensions of the scales in sample 1(n=343). The EFA suggests that the entire 21 items load on 3 factors just as classified above: social cues, cultural cues, and online cues.The remaining 19 items are translated into English as listed in Table 5

**T****able 5. Exploratory Factor Analysis Results: Sample 1 (n = 343)**

| **Factor/Item** | **Factor Loading** | **Cronbach’s**  **α** |
| --- | --- | --- |
| ***Factor 1: Social cues*** |  | 0.920 |
| Superstars and celebrities are active that day. | 0.759 |  |
| Most friends in my WeChat circle participate. | 0.854 |  |
| I feel the “Renao” (happily crowded) atmosphere. | 0.798 |  |
| Every year the SDSFs is reported as the hot event. | 0.854 |  |
| I receive many shopping strategies from Xiaohongshu, WeChat, and other social media. | 0.847 |  |
| Participants share their funny experiences. | 0.839 |  |
| ***Factor 2: Cultural cues:*** |  | 0.903 |
| I feel the SDSFs is a special day. | 0.747 |  |
| I have the ritual feeling, so I must buy something. | 0.703 |  |
| I prepare a long time for the day every year. | 0.773 |  |
| Shopping on Singles Day is a treatment for loneliness. | 0.865 |  |
| Buy! Buy! Buy! The consumerist culture is the point. | 0.784 |  |
| I am pressed for time. I have to wait for the next year if I miss the deal. | 0.763 |  |
| ***Factor 3: Online cues:*** |  | 0.899 |
| Most of China’s e-commerce companies take part in the SDSFs. | 0.717 |  |
| The red background color and red envelopes highlight the joyous and festive atmosphere. | 0.727 |  |
| The logo, discount tag, and virtual division settings make the SDSFs more informative. | 0.800 |  |
| A large number of brands, vendors, and items take part in the SDSFs. | 0.800 |  |
| Many online games increase participants’ pleasure. | 0.781 |  |
| The layout and process designed for both desktop and mobile shopping make the SDSFs more effective. | 0.756 |  |
| The sales are fair. | 0.710 |  |

**Page 16 line 484-486**

3.3 Reanalysis of measures

*confirmatory factor analysis.* Confirmatory factor analysis (CFA) was performed on sample 2 (n=343) in AMOS21.0 to validate the factor structure extracted from the previous EFA.

**Page 16 line 495-497**

*Criterion-related validity and cross-validation.* Table 5 shows the CFA results of the sample 2 (n=343). After purification once more by the sample 1 (n=343), the measurement model of the validation sample is found to be statistically significant.

**Page 14 line 498-506**

In addition, as shown in Table 6., construct and discriminant validity concerns are not found (i.e., composite reliabilities>0.7, average variance extracted (AVE)>0.5) [87]. The square root of AVE (the bold diagonal number in the table) is higher than the correlation coefficient between structures, indicating that discriminant validity is satisfied [88], as shown in Table 7.

**Table 6. Performance of the measurement scale from CFA (sample 2, n=343).**

| **Items** | **Composite Reliability** | **AVE** | **Factor  Loading** | **Critical  Ratio** | **p** |
| --- | --- | --- | --- | --- | --- |
| ***Social cues:*** | 0.926 | 0.678 |  |  |  |
| Participants share their funny experiences. |  |  | 0.838 | — | *** |
| Superstars and celebrities are active that day. |  |  | 0.77 | 16.421 | *** |
| Most friends in my WeChat circle participate. |  |  | 0.835 | 18.877 | *** |
| I feel the “*Renao*” (happily crowded) atmosphere. |  |  | 0.846 | 19.35 | *** |
| Every year the SDSFs is reported as the hot event. |  |  | 0.852 | 19.525 | *** |
| I receive many shopping strategies from *Xiaohongshu*, *WeChat*, and other social media. |  |  | 0.795 | 17.66 | *** |
| ***Cultural cues:*** | 0.906 | 0.616 |  |  |  |
| Buy! Buy! Buy! The consumerist culture is the point. |  |  | 0.83 | — | *** |
| I feel the SDSFs is a special day. |  |  | 0.711 | 14.542 | *** |
| I have the ritual feeling, so I must buy something. |  |  | 0.739 | 15.268 | *** |
| I prepare a long time for the day every year. |  |  | 0.813 | 17.655 | *** |
| Shopping on Singles Day is a treatment for loneliness. |  |  | 0.835 | 18.166 | *** |
| I am pressed for time. I have to wait for the next year if I miss the deal. |  |  | 0.772 | 16.177 | *** |
| ***Online cues:*** | 0.902 | 0.607 |  |  |  |
| A large number of brands, vendors, and items take part in the SDSFs. |  |  | 0.81 | — | *** |
| The red background color and red envelopes highlight the joyous and festive atmosphere. |  |  | 0.688 | 13.488 | *** |
| The logo, discount tag, and virtual division settings make the SDSFs more informative. |  |  | 0.824 | 17.365 | *** |
| Many online games increase participants’ pleasure. |  |  | 0.826 | 16.978 | *** |
| The layout and process designed both for desktop and mobile shopping make the SDSFs more effective. |  |  | 0.764 | 15.426 | *** |
| The sales are fair. |  |  | 0.752 | 15.102 | *** |

*** p<0.001.

**Table 7. Correlations of the measurement scale from CFA (sample 2, n=343)**

|  | Correlations | | |
| --- | --- | --- | --- |
|  | Social cues | Cultural cues | Online cues |
| Social cues | **0.823** |  |  |
| Cultural cues | 0.490 | **0.785** |  |
| Online cues | 0.411 | 0.585 | **0.779** |

The second concern is that “ the search for items in the construct development phase during the semi structured interviews is not convincing.” Our explanations are as follows:

(1) We conducted interviews with 23 participants after participating in the " China’s Singles Day Shopping Festivals " on November 11, 2019. The interview questions do not deliberately emphasize what kind of "shopping festival atmosphere" is "good", nor do they set up specific descriptions about "shopping festival atmosphere" in advance, which will effectively avoid the suspicion of excessive guidance in the interview process, so as to obtain the most authentic feelings of the respondents on "shopping festival atmosphere".

(2) According to the semi-structured questions, from the recognition of shopping festivals to the feeling of shopping festival atmosphere, this paper continuously develops the thinking of the respondents, encourages them to share shopping stories and experiences, and finally provides the original materials for the scale refinement of this paper.

(3) The preliminary items of this study came from the content collation of in-depth interviews at that time. However, with the accumulation of research findings on " China’s Singles Day Shopping Festivals " in recent years, most of the preliminary items in this study also have source basis.

**3.** The author refers to standard models in marketing (SOR model) but I miss a clear reference to the origins of these models (e.g., Woodworth, or Howard and Sheth).

**Response 3**: Thank you for your advice. This study adds review the literature on the origin and application of S-O-R model, and demonstrate and analyze the feasibility of this model as the theoretical background of this paper. In the part of theoretical basis, we also follow your suggestion and refer to Woodworth's paper on S-O-R theory. See Sec 2.1 of this study for details.

**Page 3-4 line111-142**

2.1	Theoretical foundation

The S-O-R (Stimulus-Organism-Response) theory is derived from the S-R (Stimulus-Response) theory of behavioral psychology [27]. The S-O-R model was first proposed by Woodworth, and he explained that stimuli elicit different responses depending on the state of the organism[28]. Environmental psychologists use the S-O-R paradigm to describe the external stimulus, intervention variables, and behavioral outcomes [29]. The salient feature of the S-O-R theory is that consumer decision making goes through three distinct stages, i.e. external stimuli (S), affecting individual emotional states (O), and finally triggering behavioral responses (R)[30]. S-O-R theory is widely used to study offline and online consumer psychology and behavior. Currently, popular online consumption scenarios include: Fashion e-commerce retail [31], TikTok store [32], Live streaming e-commerce [33].

Kotler (1973) defined atmosphere as an environment designed to elicit emotional and behavioral responses from customers [19]. In 1974, Mehrabian and Russell pioneered the use of the S-O-R paradigm to examine the influence of atmospheric factors on consumer behavior [29]. In the positivist paradigm (S-O-R), numerous scholars argue that atmosphere is typically external stimulus (S) that influences the consumers' internal organism (O), subsequently leads to behavioral response (R) [34-36]. In recent years, the S-O-R framework has been employed extensively to examine the online shopping atmosphere in a multitude of contexts. For example, the visual complexity of shopping websites [37] and the website informativeness and effectiveness of information content [38] have been identified as key factors. Chen and Li (2019) posited that the atmosphere of shopping festivities is the primary factor influencing consumer purchasing intentions [24]. Xie et al. (2023) investigated the influence of the atmosphere of an online shopping festival as a stimulus factor on consumers' intention to engage in continued participation [26]. In accordance with the S-O-R theory, Cheng and Chen (2023) devised a creative atmosphere scale, and its process and methodology provide a very valuable reference for this research [39]. This study puts forth the concept of shopping festival atmosphere (SFA) from the vantage point of consumption perception stimulus, with a foundation in the S-O-R framework. SFA is evidenced by the implementation of extensive product promotions, the configuration of online platforms, the incorporation of entertainment games, the facilitation of social interaction, and other forms of expression. SFA is a stimulating element (S), consumers experience the joy, stimulation, and excitement associated with shopping carnivals (O), ultimately exhibiting a tendency to engage in continuous participation (R).

**4.** The paper discusses scale development for atmosphere at the SDSF event. However, for me, it is unclear, whether this scale could also be used to measure atmosphere at other online and offline events. Usually, if you develop a standard scale, it is used to compare competitors, users, and the like. The author should more clearly explain why it is so important to develop a scale that is focused on one specific event.

**Response 4:** Thank you for pointing out this issue. According to our understanding, it is necessary to further demonstrate the academic value and practical significance of the research in this paper. Allow us to make the following explanation.

(1) “China's Singles Day Shopping Festivals” brings even more daily sales than the traditional Western “Black Friday” shopping carnival (Chen & Li, 2019), and it has been recognized as the world's It has been recognized as the world's largest and most influential online promotional activity (Xu et al., 2017, Xie et al., 2023). It is worthy of in-depth study.

(2) In the research literature on “China's Singles Day Shopping Festivals” in the past five years, “atmosphere” appears in the papers of many scholars. In the S-O-R paradigm, scholars usually take “shopping festivals atmosphere” as a stimulus variable (Zhao and Wan,2017; Shuai, Lei and Jiemin, 2018; Chen and Li, 2019; Chen and Li, 2020; Xie et al., 2023) or a moderator variable (Li et al., 2020) in the study of consumer psychology and behavior. However, there is no in-depth research on how to express “shopping festival atmosphere”.

(3) Some scholars have also studied the festive atmosphere of physical stores (Huang, 2017). The research results on “shopping festival atmosphere” are also providing new ideas for the study of offline shopping festival atmosphere in future.

(4) Online shopping festivals have entered the era of live e-commerce. There is a certain research foundation on the shopping atmosphere of “live e-commerce” (Tong, Chen, Zhou, 2023; Shi, Li, Hu, 2023). The research on the “atmosphere of live e-commerce” during online shopping festivals has yet to be explored in depth. The “shopping festivals atmosphere” scale developed in this paper is also of some significance to the research in this field.

(5) In view of the similarity of the development of global e-commerce platforms, online shopping festivals have also begun to rise in other countries (such as Cyber Hot Days in Korea, Diwali Festival in India, Black Friday, Cyber Monday in the USA, and Harbolnas in Indonesia) (Yulianto, Sisko, and Hendriana,2021). This study also provides reference value for other countries to establish online shopping festivals with the help of e-commerce platforms.

(6) Based on the above arguments, this study on “shopping festivals atmosphere” is valuable. It not only provides suggestions for online shopping platforms and offline physical stores on how to create a comfortable shopping atmosphere and stimulate their consumption desire.

In this paper, the research value of SDFS and the rationale for choosing SDFS are added to the introduction section.

**Page 2-3 line 84-95**

"SDSFs" created by Alibaba is the earliest online shopping festival in China (2009.11.11), with the highest popularity, the most extensive consumer participation and the most influential [8, 10]. The success of other online shopping festivals (e.g. JD 6.18) also draws lessons from the successful experience of SDSFs. The rapidly growing sales data of "SDSFs" over the years and the popular discussion on social platform [1, 23]. SDSFs has undergone a significant transformation, evolving from a focus on simple price promotions to the development of comprehensive marketing campaigns that prioritize the creation of an entertaining and interactive atmosphere [26]. Given the relatively standardized and homogeneous nature of e-commerce activities worldwide, the findings of the survey on Chinese e-commerce platforms can be extrapolated to a number of other platforms globally. This offers valuable insights and recommendations for other countries preparing for similar shopping festivals. Therefore, the "SDSFs" is not only a typical representative of online shopping festival, but also provides reference value for the development of similar online shopping festivals.

# Response to Reviewer 2 Comments

**1. Summary**

Thanks for reviewer’s comments and suggestions. We have answered these questions in detail and revised our manuscript carefully. We would like to express our great appreciation again for giving us the opportunity to revise our manuscript. Thank you very much for your kind consideration.

Reviewer #2: Thank you for conducting such interesting research. Although it has several advantages, there are also some issues that you should resolve before further processing.

**1. The introduction**

• The introduction provides a good overview of the importance and growth of China's Singles Day Shopping Festivals (SDSFs), highlighting their massive scale and increasing popularity.

• It identifies the lack of systematic analysis of the atmospheric elements of these major online sales events as a research gap.

• The objectives of the study, which are to develop a measurement scale for "shopping festival atmospherics" (SFA) and examine participants' perceptions of the atmospherics, are clearly stated.

But there are some Weaknesses:

• The introduction could be strengthened by providing more context on the existing research on shopping atmospherics, both in offline and online settings. This would help situate the current study within the broader literature. I suggest adding the following research to your paper to improve this section:

Shafiee, M. M., & Es-Haghi, S. M. S. (2017). Mall image, shopping well-being and mall loyalty. International Journal of Retail & Distribution Management, 45(10), 1114-1134.

Shafiee, M. M., & Bazargan, N. A. (2018). Behavioral customer loyalty in online shopping: The role of e-service quality and e-recovery. Journal of Theoretical and Applied Electronic Commerce Research, 13(1), 26-38.

Tabaeeian, R. A., & Mohammad Shafiee, M. (2023). Identifying factors affecting the motivation of games users in social networks and their impact on the user attitude and shopping intention. New Marketing Research Journal, 12(4), 51-68.

• The rationale for focusing specifically on SDSFs, rather than online shopping festivals in general, could be elaborated on further.

• Expand the literature review in the introduction to give a more comprehensive overview of the existing research on shopping atmospherics and how it has been applied in online and festival settings.

• Clearly articulate the theoretical and practical significance of developing a scale to measure SFA.

• Provide a more detailed justification for the choice of SDSFs as the context for this study.

**Response 1:** Thank you very much for your wonderful comments.

First of all, thank you for your recognition of the content introduced in this article. Secondly, thank you very much for your reminder about adding the latest research results on "shopping Festival atmosphere". Finally, I would like to thank you more for your research literature.

(1) This paper adds the research results on the atmosphere of shopping festivals in the past five years to the introduction, as shown below.

**Page1 line 40-50:**

Improving the variety of promotion categories and the interest of promotion activities are very effective promotion strategies during online shopping festival [9]. Information quality, product quality and cost savings can significantly improve consumer satisfaction with online shopping festivals [10]. Later, researchers found that the Double 11 online shopping festival was not only a promotional activity, but also an entertainment for participants. Collaboration between Chinese e-commerce platforms and merchants in terms of product promotion and atmospheric marketing strategies is key to the success of the SDSFs [9]. Xu, Wang, and Zhao (2020) posit that SDSFs creates unique shopping scenarios that can more effectively stimulate consumers' emotions and influence their shopping behaviors [11]. Hedonistic shopping has become the main motivation for consumers to participate in online shopping festivals, in addition to saving money [12].

**Page 2 line71-82**

The influence of shopping festival atmospheres on consumer behavior in SDSFs has also been the subject of research by scholars in the field. In a study conducted by Yang, Li, and Zhang (2018), it was discovered that the atmosphere surrounding SDSFs was negatively correlated with consumers' intentions to engage in sustainable consumption [23]. This atmosphere has become SDSFs most important marketing tool for stimulating consumer purchases [24]. The shopping festival atmosphere plays a positive moderating role between promotional stimulation and satisfaction, as well as between satisfaction and the intention to continue participating [25]. Despite the considerable attention that research on the atmosphere of online shopping festivals has attracted, there is still no consensus on the exact connotations of this phenomenon. There are no clear conclusions about the difference between the atmosphere of online shopping festivals and that of traditional shopping environments, and there is a lack of scientifically valid scales to describe this unique atmosphere.

(2) This paper adds the reasons for selecting ‘SDSFs’ as the research object to the introduction, as shown below.

**Page 2-3 line 84-95**

"SDSFs" created by Alibaba is the earliest online shopping festival in China (2009.11.11), with the highest popularity, the most extensive consumer participation and the most influential [8, 10]. The success of other online shopping festivals (e.g. JD 6.18) also draws lessons from the successful experience of SDSFs. The rapidly growing sales data of "SDSFs" over the years and the popular discussion on social platform [1, 23]. SDSFs has undergone a significant transformation, evolving from a focus on simple price promotions to the development of comprehensive marketing campaigns that prioritize the creation of an entertaining and interactive atmosphere [26]. Given the relatively standardized and homogeneous nature of e-commerce activities worldwide, the findings of the survey on Chinese e-commerce platforms can be extrapolated to a number of other platforms globally. This offers valuable insights and recommendations for other countries preparing for similar shopping festivals. Therefore, the "SDSFs" is not only a typical representative of online shopping festival, but also provides reference value for the development of similar online shopping festivals.

(3) The theoretical and practical significance of developing the SFA scale is partially described in the introduction section (as follows), and the detailed part is added to Section 4 of this study with the contents of 4.1 Theoretical implications and 4.2 Managerial implications.

**Page 2-3 line 97-104**

This study posit that atmospherics represents one of the most salient characteristics of large-scale online sales events. In accordance with the nomenclature of the SDSFs, the present study endeavors to encompass the comprehensive dimensions of "shopping festival atmospherics" (SFA). Additionally, as Kolter (1973) noted that the intended atmosphere differs from the perceived atmosphere, the authors also investigate participants’ perception of the atmospherics[19]. This study is expected to contribute to the literature in two ways: firstly, according to the S-O-R theory, explore the shopping festival atmosphere related to online mass sales activities, and secondly, develop a SFA (including cultural cues, social cues, and online cues) measurement scale for future research on online festival sales activities.

**Page18 line 540-554**

4.1Theoretical implications

This research has a number of theoretical implications. First, according to the S-O-R theory, which focuses on "environmental stimuli can trigger individual emotional responses", combined with previous research findings, a shopping festival atmosphere scale was developed. In recent years, with the widespread attention of scholars on online shopping atmosphere, scholars have described the connotation of shopping festival atmosphere from different perspectives [24, 26]. The research findings of this paper not only establish the concept of shopping festival atmosphere, but also expand its theoretical connotation and application.

Secondly, the SFA scale developed in this paper is different from previous measures of atmosphere because it contains three components: cultural, social, and online atmosphere, and it is described in detail. The shopping festival atmosphere not only adds the concept of "festival" to the original online shopping atmosphere, but also takes into account China's unique cultural background and the most extensive online and offline social interaction. This concept extends the application of atmosphere research to the phenomenon of "online shopping festival". The connotation of the shopping festival atmosphere identified in this study is discussed as follows:

**Page18 line 556-557; line 560-561; line 562-565**

Cultural clues refer to the particularity and sense of ritual that must be consumed in online shopping festivals. Just as Zhao and Wan (2017) found in their study that participating in SDSFs is a ritual event [35]. The Confucian values of "keeping face" and "listening to others" exert a moderating influence on the effect of the atmosphere of the online shopping festival on purchase intention [24]. The happily crowded environment (“Renao” in the Chinese language) is a positive atmospheric cue for Chinese participants.

**Page18-19 line 568-576**

Social cues mean that participants actively participate in the most extensive topic communication and knowledge sharing during the SDSFs, both offline and online. The extensive social interaction on the shopping day (such as topic discussion, shopping communication, experience sharing, etc.) has gradually become the biggest feature of Singles Day shopping day [9, 25]. Through online and offline advertising, single consumers can establish a sense of identity with Singles Day [35]. Furthermore, Chen and Li (2020) demonstrated that the festive atmosphere exerts an influence on consumers, which is shaped by the social medias and the people around them [9]. All of these social environments should ultimately result in a crowded atmosphere that is safe, secure and happy. Otherwise, participants may experience negative emotions and misbehave [15, 18].

**Page19 line 578-587**

Online clue is the evaluation of the comprehensive service of the network platform by the consumers participating in the online shopping festival. As an online sales event, the SDSF must change for the special day. This study found that in addition to the traditional online climate of information, efficiency and entertainment, the fair climate was also a new feature perceived by participants. They need very good deals [25]. They don't want to be cheated by artificially inflated prices and they don't want to go to great lengths to play the coupon game. In addition, the authors found that size itself is an attribute of online atmosphere. Studies by Tzeng, Ert, Jo et al. (2020) show that product information and product quality are prerequisites for consumers to feel satisfied after participating in online shopping festivals [10]. The website informativeness and effectiveness of information content can help to improve consumer satisfaction and purchase intention [38].

**Page19 line 592-605**

4.2 Managerial implications

This study has established a reliable scale for measuring the "shopping festival atmosphere," and has also provided managers with a tool for objectively understanding consumers' perception of the shopping festival atmosphere. The results of this study have significant implications for the practice of management, which can be grouped into many areas: Firstly, managers are able to utilize this scale to ascertain whether the shopping festival atmosphere has been successfully created, particularly when the SFA is low. Subsequently, managers are able to modify their strategies in accordance with the aforementioned findings. Secondly, the utilization of cultural, social and online cues can also serve as a valuable means of evidence for managers seeking to establish a shopping festival atmosphere. Thirdly, these cues can be integrated with actual circumstances to develop innovative designs that enhance consumers' purchase intention during the shopping festival. Ultimately, the findings of this study offer valuable insights that can inform the successful implementation of online shopping festivals in other countries, particularly in light of the growing prevalence of such events in China and the global convergence of e-commerce platforms.

**2. Literature Review:**

• The literature review covers the key concepts and theories relevant to the study, including the definition of atmospherics and its application in retail and festival settings.

• The review discusses the existing research on the success factors and motivations of consumers for participating in online shopping festivals, providing useful background information.

• However, the literature review could be more comprehensive, incorporating a wider range of studies on shopping atmospherics, both in offline and online contexts. Please use previous mentioned sources to improve this section.

• The review could also delve deeper into the specific atmospheric cues that have been identified in the literature for offline and online shopping environments, as well as festival settings.

• Expand the literature review to include a more thorough examination of the broader research on shopping atmospherics, highlighting the key dimensions and cues that have been identified in previous studies.

• Discuss how the atmospherics of online shopping environments and festival settings may differ from traditional offline retail settings, and the implications for the current study.

• Identify any gaps or limitations in the existing literature that the current study aims to address.

I also suggest using and adding the following sources to update your sources in this section:

Shafiee M. M., Yavari, Z. & Ghorbanian, P. (2015). Ranking of Selected Convenience Stores of Isfahan Based on Store Image Dimensions with Group AHP Technique. Journal of Operational Research and Its Applications, 12(46), 35-47.

Ghorbanian, P., Yavari, Z., & Mohammad Shafiee, M. (2015). Analysis of retailer equity based on selected store image dimensions (Case study: Refah, CityCenter (HyperStar) & Kowsar stores). New Marketing Research Journal, 5(3), 143-160.

Mohammad Shafiee, M., & Ahghar Bazargan, N. (2016). Electronic trust of customers to online stores with a risk reduction approach. Journal of Karafan, 6(10), 113-122.

Mohammad Shafiee, M., Bazargan, N. A., & Kazeminia, A. (2017). Modeling Customer Electronic Trust in Online Stores: A Risk Reduction Approach. 11th International Conference on e-Commerce in Developing Countries (ECDC), April, Isfahan, Iran.

Nurani, M., Rezaei Dolatabadi, H., & Mohammad Shafiee, M. (2021). Designing a store brand competitiveness model based on environmental stimuli In chain stores. Journal of Business Management Perspective, 19(44), 13-40.

Nurani Kutenaee, M., Rezaei Dolatabadi, H., & Mohammad Shafiee, M. (2021). Modeling the Competitiveness of a Store Brand based on the pattern of Environmental Estimuli in Chain Stores: A mixed approach. Management Research in Iran, 25(2), 151-182.

**Response 2:** Your suggestions for extending the literature review are greatly appreciated, especially for suggesting that this study add "How the atmosphere of the online shopping environment and the festive environment differs from the traditional offline retail environment".

Through a systematic regression of previous literature, the differences between the atmosphere of online shopping environment and festive environment and traditional offline retail environment include the following aspects: (1) The traditional offline retail environment can be created through space design, color, music, smell and product display. (2) The atmosphere of the online shopping environment is mostly created through visual effects and website navigation. (3) The festival atmosphere of online shopping not only has eye-catching visual effects and efficient website navigation, but also increases social interaction, the excitement of game entertainment, the sense of identity of group participation and the tension of flash sale.

For the literature review section, we have focused on two pieces of literature provided by you.

**Page 3 line 125-128**

In the positivist paradigm (S-O-R), numerous scholars argue that atmosphere is typically external stimulus (S) that influences the consumers' internal organism (O), subsequently leads to behavioral response (R)[34-36].

**Page 9 line 358-359**

It is consistent with the research conclusion of Shafiee and Bazargan (2018) [76] in shopping loyalty.

34.Shafiee MM, Es-Haghi SMS. Mall image, shopping well-being and mall loyalty. International Journal of Retail & Distribution Management 2017; 45:1114-1134. <https://doi.org/10.1108/ijrdm-10-2016-0193>

76. Shafiee MM, Bazargan NA. Behavioral Customer Loyalty in Online Shopping: The Role of E-Service Quality and E-Recovery. Journal of Theoretical and Applied Electronic Commerce Research. 2018; 13:26-38. <https://doi.org/10.4067/s0718-18762018000100103>

(1) Based on relevant literature from the past five years, we have added more extensive research results on offline and online shopping atmospheres. The additions and modifications to the literature review are shown below.

**Page 4 line162-164**

2.3 Atmospherics of physical stores

In his seminal work, Kotler (1973) posited that the store atmosphere encompasses four dimensions: visual, auditory, tactile, and olfactory [19].

**Page 5 line172-173**

Similarly, the store atmosphere can be divided into three dimensions: social categories (3 items), physical categories (3 items), and ambient features (2 items) [42].

**Page 5 line189-190**

There have been many interesting findings on the study of store atmosphere on consumer behavior.

**Page 5 line193-200**

In young shoppers' sections and pop-up stores, playing modern and stylish music can increase their desire to stay in the mall [50]. In global apparel retailer's stores, customers in a non-English speaking country are more likely to buy when music is played in English [53]. The olfactory congruence of a shopping mall has a positive effect on bridging the mall atmosphere and brand image [54]. In young shoppers' sections and pop-up stores, playing modern and stylish music can increase their desire to stay in the mall [31]. The visual atmosphere of a coffee shop can have an impact on consumers' expectations of different coffee flavors [55].

2.4 Atmospherics of online stores

**Page 6 line 224-226**

Some scholars differentiate between the various elements that comprise the atmosphere of online stores, including information, navigation quality, as well as platform design [60, 61].

**Page 6 line 236-243**

Ettis (2017) found that the blue-hues background of an online store induces more consumers’flow (Enjoyment and concentration) than yellow[65]. The unique shopping atmosphere of e-commerce live streaming is reflected in three aspects: bullet screen, guide information as well as parasocial interaction [66]. In live streaming e-commerce, participants generate high arousal when they are in an atmosphere with complex background images and fast-paced background music, and fast-paced music seems to better meet the needs of consumers [33].

2.6.1 cultural cues

**Page 7 line 285-286**

Consuming during the “SDSFs”is considered a ritualistic activity [35].

2.6.2 social cues

**Page 8 line 330-335**

The atmosphere of participation in the online shopping festival will affect consumers through the medium of social interaction with the media and with other people [24]. Extensive social interaction online and offline is also a major stimulus for a large number of consumers to participate in the SDSFs [9, 25]. The social interaction atmosphere will stimulate the excitement of customers, thus enhancing the intention of consumers to continue to participate in the SDSFs [26].

2.6.3 physical cues

**Page 9 line244-346**

In order to enhance customer purchasing behavior, numerous retailers dedicate a considerable amount of time and resources to the implementation of distinctive merchandise displays at festivals, with the objective of attracting potential customers [75]. The strong technical and service support provided by Alibaba makes consumers feel the“fairness atmospherics” of the China’s Singles Day Shopping Festivals [25].

2.6.4 online cues

**Page 9 line 356-359**

An aesthetically pleasing online store design and intuitive navigation facilitate consumer engagement and enhance the shopping experience on Singles' Day [35]. It is consistent with the research conclusion of Shafiee and Bazargan (2018) [76] in shopping loyalty.

**Page 9 line 361-363**

The strong technical and service support provided by Alibaba makes consumers feel the“fairness atmospherics” of the China’s Singles Day Shopping Festivals [25].

(2) Based on previous research results, the key dimensions and cues of “shopping festival atmosphere” that have been further identified include: cultural cues, social cues, physical cues, and online cues.

There are a few scattered studies in the literature on festival atmospherics and their effects on consumer behavior. However, we collect relevant references from three domains: festival shopping (and holiday shopping), shopping festivals, and large online promotions. Combined with the above review, we identify four categories of festival atmospheric cues: cultural cues, social cues, physical cues, and online cues.

The modifications and additions to the ‘cultural cues, social cues, physical cues, and online cues’ section have been explained above. See section 2.6 in this study for details.

**3. Methodology:**

• The study employs a rigorous, multi-step approach to scale development, including both qualitative and quantitative methods.

• The use of multiple data sources (literature review, focus groups, and online survey) to generate and refine the scale items is a strength.

• The validation of the scale through confirmatory factor analysis is appropriate and well-executed.

• But the methodology section could provide more details on the sample characteristics and data collection procedures for the quantitative phase.

• The rationale for the specific statistical techniques used (e.g., exploratory factor analysis, confirmatory factor analysis) could be elaborated on further.

• Expand the methodology section to include more details on the sample composition, data collection methods, and data analysis procedures used in the quantitative phase of the study.

• Provide a clearer explanation of the decision-making process behind the selection of the statistical techniques employed.

• Consider including information on the reliability and validity assessments conducted for the final SFA scale.

**Response 3:** We are honored to see your high evaluation of this research. In response to your suggestions, we have made the following explanations and modifications:

(1) Details of the data collection procedure

(2) Data characteristics (mean and variance: two sets of samples)

(3) Exploratory factor analysis, verify the basic principle of factor analysis

(4) Methodology and scale development process

(5) Information on the reliability and validity assessment of the final SFA scale

(1) Details of the data collection procedure

We designed the questionnaire and entrusted it to the largest questionnaire service company in China—wjx.cn (https://www.wjx.cn/) for questionnaire collection. The detailed process of questionnaire collection is as follows:

1) The authors designed the questionnaire according to the purpose of the study.

2) Using the service function of the wjx.cn (https://www.wjx.cn/), the questionnaire can be directly read and filled out on the mobile phone.

3）Entrust wjx.cn (https://www.wjx.cn/) (the largest questionnaire service platform in China) to sign the service agreement to determine the demographic attributes of the questionnaire collection, the number of questionnaires and the reward of the questionnaire.

4）According to the reading of the survey instructions, the respondents decide whether to fill in the questionnaire voluntarily. They can scan the QR code or open the link to fill in the questionnaire through their mobile phones, and receive the reward after completing the questionnaire.

5）Finally, the collected questionnaires were preprocessed, and the unreasonable questionnaires such as incomplete questionnaires and extreme answers were eliminated. The final valid questionnaires were used for data statistical analysis.

We have added the details of the data collection to the revised manuscript.

**Page 12 line 430-434**

According to the survey instructions, the respondents decide whether to fill in the questionnaire voluntarily. They can scan the QR code or open the link to fill in the questionnaire through their mobile phones, and receive the reward after completing the questionnaire. The total sample size is 738. After deleting uncertain samples (e.g., incomplete answers, all the same answers, minors), the valid sample size is 686 (Six samples involving minors were deleted from the original data).

(2) Data characteristics (mean and variance: two sets of samples)

**Page 12 line 435-440**

As shown in Table 3., more than 98% of the respondents have participated in SDSFs, while approximately 80% participated in SDSFs almost every time. Approximately 75% of the respondents are from the young generation aged 20 to 40. However, there are still a quarter of the respondents who are middle-aged. In addition, more than half of the respondents have frequent chat experiences (5 to 19) during SDSFs. The demographics show that SDSFs is indeed an influential sales event attracting a large participant population.

**Table 3. Descriptive Statistics of Respondents (Sample 1 and Sample 2)**

| **Variable** | | **Sample 1 (n = 343)** | | **Sample 2 (n = 343)** | |
| --- | --- | --- | --- | --- | --- |
| Gender | Male | 157 | 45.77% | 166 | 48.40% |
|  | Female | 185 | 53.94% | 177 | 51.60% |
| Age | 19 to 25 | 80 | 23.32% | 86 | 25.07% |
|  | 26 to 30 | 73 | 21.28% | 75 | 21.87% |
|  | 31 to 40 | 110 | 32.07% | 98 | 28.57% |
|  | 41 to 50 | 65 | 18.95% | 71 | 20.70% |
|  | 51 to 60 | 14 | 4.08% | 12 | 3.50% |
|  | Greater than 61 | 1 | 0.29% | 1 | 0.29% |
| Education level | Middle school | 24 | 7.00% | 20 | 5.83% |
|  | High school | 47 | 13.70% | 47 | 13.70% |
|  | Bachelor’s degree | 219 | 63.85% | 221 | 64.43% |
|  | Graduate degree | 49 | 14.29% | 53 | 15.45% |
|  | Post-graduate degree | 4 | 1.17% | 2 | 0.58% |
| Occupation | Working | 216 | 62.97% | 218 | 63.56% |
|  | Student | 59 | 17.20% | 61 | 17.78% |
|  | Freelancer | 42 | 12.24% | 36 | 10.50% |
|  | Retired | 1 | 0.29% | 1 | 0.29% |
|  | Other | 25 | 7.29% | 27 | 7.87% |
| Monthly income | Less than CNY 1000 | 31 | 9.04% | 41 | 11.95% |
|  | CNY 1001 to 3000 | 39 | 11.37% | 37 | 10.79% |
|  | CNY 3001 to 5000 | 55 | 16.03% | 47 | 13.70% |
|  | CNY 5001 to 8000 | 115 | 33.53% | 111 | 32.36% |
|  | CNY 8001 to 15000 | 66 | 19.24% | 76 | 22.16% |
|  | CNY 15001 to 20000 | 31 | 9.04% | 23 | 6.71% |
|  | Greater than CNY 20001 | 6 | 1.75% | 8 | 2.33% |
| Participation counts | Every time | 168 | 48.98% | 142 | 41.40% |
|  | Almost every time | 113 | 32.94% | 141 | 41.11% |
|  | Several times recently | 58 | 16.91% | 54 | 15.74% |
|  | Never | 4 | 1.17% | 6 | 1.75% |
| Chat counts during SDSFs | Greater than 20 | 22 | 6.41% | 20 | 5.83% |
|  | 10 to 19 | 59 | 17.20% | 55 | 16.03% |
|  | 5 to 9 | 139 | 40.52% | 135 | 39.36% |
|  | 1 to 4 | 116 | 33.82% | 126 | 36.73% |
|  | Never | 7 | 2.04% | 9 | 2.62% |

**Page 13 line 453-460**

In order to ensure the scientific and normative nature of the research, the collected data can be randomly divided into two parts. According to the research method and process of Dimitrova, Ilieva, and Stanev (2022) , EFA and CFA were performed on the two parts of data respectively [80]. The proportions of gender, age, income, education, occupation, participation counts, and chat counts were maintained in the subsample. The demographic characteristics of the participants in the two randomly grouped sub-samples meet the requirements of the independence chi-square test. The numerical characteristics (mean and standard deviation) of the survey sample are shown in Table 4.

**Table 4 The measure of the sample data (n=686)**

|  | **Sample 1 (n = 343)** | | **Sample 2 (n = 343)** | |
| --- | --- | --- | --- | --- |
| **Factor/Item** | **Mean** | **Standard Deviations** | **Mean** | **Standard Deviations** |
| Superstars and celebrities are active that day. | 5.379 | 1.470 | 5.466 | 1.416 |
| Most friends in my WeChat circle participate. | 5.207 | 1.469 | 5.440 | 1.406 |
| I feel the “Renao” (happily crowded) atmosphere. | 5.122 | 1.574 | 5.239 | 1.457 |
| Every year the SDSFs is reported as the hot event. | 5.172 | 1.542 | 5.309 | 1.508 |
| I receive many shopping strategies from Xiaohongshu, WeChat, and other social media. | 5.052 | 1.594 | 5.233 | 1.531 |
| Participants share their funny experiences. | 5.050 | 1.597 | 5.265 | 1.466 |
| I feel the SDSFs is a special day. | 4.606 | 1.754 | 4.633 | 1.832 |
| I have the ritual feeling, so I must buy something. | 4.962 | 1.648 | 4.921 | 1.612 |
| I prepare a long time for the day every year. | 4.799 | 1.718 | 4.831 | 1.790 |
| Shopping on Singles Day is a treatment for loneliness. | 5.012 | 1.656 | 5.149 | 1.790 |
| Buy! Buy! Buy! The consumerist culture is the point. | 5.096 | 1.649 | 5.058 | 1.657 |
| I am pressed for time. I have to wait for the next year if I miss the deal. | 5.073 | 1.589 | 5.082 | 1.750 |
| Most of China’s e-commerce companies take part in the SDSFs. | 5.452 | 1.434 | 5.536 | 1.468 |
| The red background color and red envelopes highlight the joyous and festive atmosphere. | 5.531 | 1.456 | 5.504 | 1.535 |
| The logo, discount tag, and virtual division settings make the SDSFs more informative. | 5.052 | 1.661 | 5.105 | 1.602 |
| A large number of brands, vendors, and items take part in the SDSFs. | 5.003 | 1.563 | 5.047 | 1.616 |
| Many online games increase participants’ pleasure. | 5.216 | 1.593 | 5.210 | 1.570 |
| The layout and process designed for both desktop and mobile shopping make the SDSFs more effective. | 5.239 | 1.599 | 5.359 | 1.575 |
| The sales are fair. | 5.044 | 1.575 | 5.090 | 1.591 |

(3) Exploratory factor analysis, verify the basic principle of factor analysis

Firstly，Exploratory factor analysis (EFA): 1) The basic structure was sought to solve the problem of strong correlation between variables in multivariate statistical analysis; 2) Data reduction; 3) Developmental measurement scale. Secondly, Confirmatory factor analysis (CFA):1) Verify the dimension or orientation of the scale and determine the most effective factor structure; 2) Validation factor hierarchy; 3) The reliability and validity of the scale were assessed.

EFA and CFA are necessary tools for scale development, and the specific principles and processes are also explained in the article.

**Page14 Line 463-469**

The present study employs exploratory factor analysis (EFA) with a varimax rotation to determine the dimensions of the scales in sample1(n=343). Items with factor loadings lower than 0.4 or cross-loaded on more than one factor are eliminated to ensure each attribute loads only on one factor [81]. In addition, the internal reliability of each factor is measured using Cronbach’s alpha. Churchill (1979) suggests that a low alpha coefficient indicates the item has a low impact on the measurement of the construct [78]. Therefore, factors with Cronbach’s alphas of less than 0.7 are eliminated [82].

**Page16 Line 486-494**

Confirmatory factor analysis (CFA) was performed on sample 2 (n=343) in AMOS21.0 to validate the factor structure extracted from the previous EFA. The degrees of freedom ratio (i.e., χ^2/df) is 3.921 [84], comparative fit index (CFI) is 0.905, and non-normed fit index (NNFI) is 0.910[85]. The root mean square error of approximation (RMSEA) is 0.092. However, the established criteria are CFI>0.90, NNFI>0.95, and RMSEA<0.08 [84, 86]. The CFA results do not meet the criteria very well. However, after deleting the item “Most of China’s e-commerce companies take part in the SDSFs” (online cues), the results improve. It can be understood that this item is highly correlated with the item “a large number of brands, vendors, and items take part in the SDSFs”. Ultimately, a total of 3 dimensions and 18 attributes remains in SFA.

(4) Methodology and scale development process

Based on previous relevant literature (Cheng and Chen, 2023; Churchill, 1979) and reference books on scale development (DeVellis, 2021), combined with the research topic, this paper forms a set of scientifically standardized ‘shopping holiday atmosphere’ scale development processes, which provide a clearer explanation of the decision-making process for selecting statistical techniques.

We have drawn the method and process of scale development into a table. For details, please refer to Page9 Table 1.

**Page9 Line 366-374**

The present study employs the conventional procedures for developing measures proposed by Churchill (1979) [23]. In accordance with Churchill's (1979) eight-step measurement development paradigm, the present study proposes that scale development can be divided into three principal stages: (1) initial item generation, (2) purification of the measurement scale, and (3) reanalysis of measures. Additionally, this study draws upon the methodology and research process of Cheng and Chen (2023) in formulating the creative atmosphere scale [21], as detailed in Table 1 below

**Table 1. The Process of the SFA Scale Development**

| **Phase** | **Description** | **Results** |
| --- | --- | --- |
| 1. Initial item  generation | 1. Literature Review | Online shopping festival, atmosphere |
|  | 2. In-depth interview (n = 23) | Consumers participating in the SDSF |
|  | 3. Content analysis | Produce to 4 categories. |
|  | 4. Content validity (2 experts) | Final results: 43 generated items. |
| 2. Purifying the measurement scale | 1. Refinement of instruments  (panel of experts to obtain content validity)  2. Data collection  3. Purifying the measurement  (Sample 1, n = 343), used EFA factor loadings and test of reliability (Cronbach’s $\alpha$) to reduce scale | Reduced "Physical cues";  Removed and optimized 24 items that were not suitable for "SFA";  Final results: 19 items belonging to 3 dimensions: 1) cultural cues (6 items), 2) social cues (6 items), and 3) online cues (7 items). |
| 3. Reanalysis  of measures | 1. Data collection (Sample 2,  n = 343); conducted CFA with  Sample 2 using three dimensions.  2. Criterion-related validity  3. Construct validity analysis | Reduce one item;  CFA confirms strength of  the scale with 18 items  (three dimensions):  1) cultural cues (6 items);  2) social cues (6 items);  3) online cues (6 items). |

Note: SDSF=China’s Singles Day Shopping Festivals; SFA= Shopping festival atmospherics;

EFA = exploratory factor analysis; CFA = confirmatory factor analysis.

(5) Information on the reliability and validity assessment of the final SFA scale

**Page13 Line 454-456**

In order to ensure the scientific and normative nature of the research, the collected data can be randomly divided into two parts. According to the research method and process of Dimitrova, Ilieva, and Stanev (2022), EFA and CFA were performed on the two parts of data respectively [80].

**Page14-17 Line 462-506**

The information on the reliability and validity of the final SFA scale has been discussed in detail in sections 3.2 and 3.3, and the relevant data can be found in Tables 5, 6 and 7.

Purifying the measurement. The present study employs exploratory factor analysis (EFA) with a varimax rotation to determine the dimensions of the scales in sample1(n=343). Items with factor loadings lower than 0.4 or cross-loaded on more than one factor are eliminated to ensure each attribute loads only on one factor [81]. In addition, the internal reliability of each factor is measured using Cronbach’s alpha. Churchill (1979) suggests that a low alpha coefficient indicates the item has a low impact on the measurement of the construct [78]. Therefore, factors with Cronbach’s alphas of less than 0.7 are eliminated [82].

The EFA suggests that the entire 21 items load on 3 factors just as classified above: social cues, cultural cues, and online cues. The first factor consists of 6 items with a Cronbach’s alpha of 0.920. However, the item “Alibaba's shopping theme gala” is deleted from analysis due to its cross-loading on another factor. The second factor consists of 6 items with a Cronbach’s alpha of 0.903. However, the item “Impulsive purchasing is tolerated that day” is deleted from analysis due to its cross-loading on another factor. In addition, the third factor consists of 7 items with a Cronbach’s alpha of 0.899. By using Statistical Package for Social Sciences 21.0, the appropriateness of factor analysis is verified. The Kaiser-Meyer-Olkin (KMO) value is 0.902, which is expressed as “marvelous” (statistic>0.9) by Kaiser (1974) [83]. Bartlett’s test of sphericity yields 4452.192 (p<0.001), indicating the factor analysis is proper. The remaining 19 items are translated into English as listed in Table 5. The items include factor 1 (social cues, 6 items), factor 2 (cultural cues, 6 items), and factor 3 (online cues, 7 items). The Cronbach’s alpha coefficients for individual SFA factors range from 0.899 to 0.920, indicating highly reliable results.

**Table 5. Exploratory Factor Analysis Results: Sample 1 (n = 343)**

| **Factor/Item** | **Factor Loading** | **Cronbach’s**  **α** |
| --- | --- | --- |
| ***Factor 1: Social cues*** |  | 0.920 |
| Superstars and celebrities are active that day. | 0.759 |  |
| Most friends in my WeChat circle participate. | 0.854 |  |
| I feel the “Renao” (happily crowded) atmosphere. | 0.798 |  |
| Every year the SDSFs is reported as the hot event. | 0.854 |  |
| I receive many shopping strategies from Xiaohongshu, WeChat, and other social media. | 0.847 |  |
| Participants share their funny experiences. | 0.839 |  |
| ***Factor 2: Cultural cues:*** |  | 0.903 |
| I feel the SDSFs is a special day. | 0.747 |  |
| I have the ritual feeling, so I must buy something. | 0.703 |  |
| I prepare a long time for the day every year. | 0.773 |  |
| Shopping on Singles Day is a treatment for loneliness. | 0.865 |  |
| Buy! Buy! Buy! The consumerist culture is the point. | 0.784 |  |
| I am pressed for time. I have to wait for the next year if I miss the deal. | 0.763 |  |
| ***Factor 3: Online cues:*** |  | 0.899 |
| Most of China’s e-commerce companies take part in the SDSFs. | 0.717 |  |
| The red background color and red envelopes highlight the joyous and festive atmosphere. | 0.727 |  |
| The logo, discount tag, and virtual division settings make the SDSFs more informative. | 0.800 |  |
| A large number of brands, vendors, and items take part in the SDSFs. | 0.800 |  |
| Many online games increase participants’ pleasure. | 0.781 |  |
| The layout and process designed for both desktop and mobile shopping make the SDSFs more effective. | 0.756 |  |
| The sales are fair. | 0.710 |  |

*confirmatory factor analysis.* Confirmatory factor analysis (CFA) was performed on sample 2 (n=343) in AMOS21.0 to validate the factor structure extracted from the previous EFA. The degrees of freedom ratio (i.e., $\chi^{2}/df$) is 3.921 [84], comparative fit index (CFI) is 0.905, and non-normed fit index (NNFI) is 0.910[85]. The root mean square error of approximation (RMSEA) is 0.092. However, the established criteria are CFI>0.90, NNFI>0.95, and RMSEA<0.08 [84, 86]. The CFA results do not meet the criteria very well. However, after deleting the item “Most of China’s e-commerce companies take part in the SDSFs” (online cues), the results improve. It can be understood that this item is highly correlated with the item “a large number of brands, vendors, and items take part in the SDSFs”. Ultimately, a total of 3 dimensions and 18 attributes remains in SFA.

*Criterion-related validity and cross-validation.* Table 5 shows the CFA results of the sample 2 (n=343). After purification once more by the sample 1 (n=343), the measurement model of the validation sample is found to be statistically significant. The results indicate a good model fit to the data, $\chi^{2}/df$=2.927, p<0.001, CFI=0.939, TLI=0.926, and RMSEA=0.075. In addition, as shown in Table 6., construct and discriminant validity concerns are not found (i.e., composite reliabilities>0.7, average variance extracted (AVE)>0.5) [87]. The square root of AVE (the bold diagonal number in the table) is higher than the correlation coefficient between structures, indicating that discriminant validity is satisfied [88], as shown in Table 7.

**Table 6. Performance of the measurement scale from CFA (sample 2, n=343).**

| **Items** | **Composite Reliability** | **AVE** | **Factor  Loading** | **Critical  Ratio** | **p** |
| --- | --- | --- | --- | --- | --- |
| ***Social cues:*** | 0.926 | 0.678 |  |  |  |
| Participants share their funny experiences. |  |  | 0.838 | — | *** |
| Superstars and celebrities are active that day. |  |  | 0.77 | 16.421 | *** |
| Most friends in my WeChat circle participate. |  |  | 0.835 | 18.877 | *** |
| I feel the “*Renao*” (happily crowded) atmosphere. |  |  | 0.846 | 19.35 | *** |
| Every year the SDSFs is reported as the hot event. |  |  | 0.852 | 19.525 | *** |
| I receive many shopping strategies from *Xiaohongshu*, *WeChat*, and other social media. |  |  | 0.795 | 17.66 | *** |
| ***Cultural cues:*** | 0.906 | 0.616 |  |  |  |
| Buy! Buy! Buy! The consumerist culture is the point. |  |  | 0.83 | — | *** |
| I feel the SDSFs is a special day. |  |  | 0.711 | 14.542 | *** |
| I have the ritual feeling, so I must buy something. |  |  | 0.739 | 15.268 | *** |
| I prepare a long time for the day every year. |  |  | 0.813 | 17.655 | *** |
| Shopping on Singles Day is a treatment for loneliness. |  |  | 0.835 | 18.166 | *** |
| I am pressed for time. I have to wait for the next year if I miss the deal. |  |  | 0.772 | 16.177 | *** |
| ***Online cues:*** | 0.902 | 0.607 |  |  |  |
| A large number of brands, vendors, and items take part in the SDSFs. |  |  | 0.81 | — | *** |
| The red background color and red envelopes highlight the joyous and festive atmosphere. |  |  | 0.688 | 13.488 | *** |
| The logo, discount tag, and virtual division settings make the SDSFs more informative. |  |  | 0.824 | 17.365 | *** |
| Many online games increase participants’ pleasure. |  |  | 0.826 | 16.978 | *** |
| The layout and process designed both for desktop and mobile shopping make the SDSFs more effective. |  |  | 0.764 | 15.426 | *** |
| The sales are fair. |  |  | 0.752 | 15.102 | *** |

*** p<0.001.

**Table 7. Correlations of the measurement scale from CFA (sample 2, n=343)**

|  | Correlations | | |
| --- | --- | --- | --- |
|  | Social cues | Cultural cues | Online cues |
| Social cues | **0.823** |  |  |
| Cultural cues | 0.490 | **0.785** |  |
| Online cues | 0.411 | 0.585 | **0.779** |

**4. Results and Discussion:**

• The results section presents the key findings from the scale development and validation process in a clear and concise manner.

• The discussion section effectively interprets the findings, highlighting the theoretical and practical implications of the study.

• The identification of novel atmospheric cues, such as the "happily crowded environment" and "fair shopping environment", is a valuable contribution.

**Response 4:** We are very grateful to the review experts for their affirmation of the research results and contributions of the article. The modifications and additions to “Results and Discussion” have been responded to in detail in “Response 1”.

**5 Weaknesses:**

• The discussion could be strengthened by more explicitly connecting the findings to the existing literature on shopping atmospherics and festival settings.

• The limitations of the study and directions for future research could be expanded upon.

• Enhance the discussion by drawing stronger links between the identified SFA dimensions and cues and the broader literature on shopping atmospherics.

• Discuss the potential cultural and contextual factors that may have influenced the emergence of the novel atmospheric cues.

• Expand the limitations section to acknowledge the study's boundaries and provide more detailed recommendations for future research.

**Response 5:** Thank you again for your valuable comments. According to our understanding, this article has been modified and adjusted as follows:

(1) In addition to the discussion of the original research results, a comparison with the conclusions of previous studies has been added to highlight the value of this study. We also enhance the discussion by drawing stronger links between the identified SFA dimensions and cues (Cultural clues; Social cues; Online cues).

The content added to compare with the conclusions of previous studies is shown below.

**Page18-19 Line 539-589**

4.1Theoretical implications

This research has a number of theoretical implications. First, according to the S-O-R theory, which focuses on "environmental stimuli can trigger individual emotional responses", combined with previous research findings, a shopping festival atmosphere scale was developed. In recent years, with the widespread attention of scholars on online shopping atmosphere, scholars have described the connotation of shopping festival atmosphere from different perspectives [24, 26]. The research findings of this paper not only establish the concept of shopping festival atmosphere, but also expand its theoretical connotation and application.

Secondly, the SFA scale developed in this paper is different from previous measures of atmosphere because it contains three components: cultural, social, and online atmosphere, and it is described in detail. The shopping festival atmosphere not only adds the concept of "festival" to the original online shopping atmosphere, but also takes into account China's unique cultural background and the most extensive online and offline social interaction. This concept extends the application of atmosphere research to the phenomenon of "online shopping festival". The connotation of the shopping festival atmosphere identified in this study is discussed as follows:

Cultural clues refer to the particularity and sense of ritual that must be consumed in online shopping festivals. SDSF was initiated from Singles Day, an unofficial festival. However, the festival creates a custom for the SDSF. It is held on November 11 every year. This is a strong cultural hint in many participants’ eyes. They treat SDSF as a special day. They have the custom or habit of accomplishing what must be done. Just as Zhao and Wan (2017) found in their study that participating in SDSFs is a ritual event [35]. In addition, they need to prepare in advance for these “must-do” behaviors. On the other hand, the consumerist culture is booming, associated with the SDSF in China. The Confucian values of "keeping face" and "listening to others" exert a moderating influence on the effect of the atmosphere of the online shopping festival on purchase intention [24]. The happily crowded environment (“Renao” in the Chinese language) is a positive atmospheric cue for Chinese participants. This type of cultural cue gives participants a symbolic link between the SDSF and the “Buy! Buy! Buy!” slogan.

Social cues mean that participants actively participate in the most extensive topic communication and knowledge sharing during the SDSFs, both offline and online. The extensive social interaction on the shopping day (such as topic discussion, shopping communication, experience sharing, etc.) has gradually become the biggest feature of Singles Day shopping day [9, 25]. Through online and offline advertising, single consumers can establish a sense of identity with Singles Day [35]. Furthermore, Chen and Li (2020) demonstrated that the festive atmosphere exerts an influence on consumers, which is shaped by the social medias and the people around them [9]. All these social environments should ultimately form a safe, secure, and happily crowded atmosphere. Otherwise, participants might have negative emotions and misbehave (Lennon et al., 2011; Swilley & Goldsmith, 2013).

Online cues are the evaluation of the comprehensive service of the network platform by the consumers participating in the online shopping festival. As an online sales event, the SDSF must change for the special day. This study found that in addition to the traditional online climate of information, efficiency and entertainment, the fair climate was also a new feature perceived by participants. They need very good deals [25]. They don't want to be cheated by artificially inflated prices and they don't want to go to great lengths to play the coupon game. In addition, the authors found that size itself is an attribute of online atmosphere. Studies by Tzeng, Ert, Jo et al. (2020) show that product information and product quality are prerequisites for consumers to feel satisfied after participating in online shopping festivals [10]. The website informativeness and effectiveness of information content can help to improve consumer satisfaction and purchase intention [38]. Participants perceive the promotion scale as they expect to have more chances to obtain what they actually want with lower prices than ordinary and more choices to avoid constraints on discounted items they want, and ultimately to achieve more benefits from the SDSF.

(2) Discuss the potential cultural and contextual factors that may have influenced the emergence of the novel atmospheric cues.

This study also explores the potential cultural and contextual factors of China's Singles' Day online shopping carnival, and explains them in the context of previous relevant research, as shown below.

Cultural clues refer to the particularity and sense of ritual that must be consumed in online shopping festivals. Just as Zhao and Wan (2017) found in their study that participating in SDSFs is a ritual event [35]. The Confucian values of "keeping face" and "listening to others" exert a moderating influence on the effect of the atmosphere of the online shopping festival on purchase intention [24]. Xu et al. (2017) attributed the success of SDSFs to the herd effect [8]. The happily crowded environment (“Renao” in the Chinese language) is a positive atmospheric cue for Chinese participants. In summary, cultural cues are intended to make shopping festivals special days.

(3) Add the section "Limitations and avenues for future research". This section acknowledges the study's boundaries and provide more detailed recommendations for future research.

**Page19-20 line 605-633**

5. Limitations and future research

In summary, this study developed a "shopping festival atmosphere" measurement scale and extracted some novel atmosphere cues from the SDSFs. Given the dearth of research in this field, however, some limitations were also identified that need to be addressed and considered.

Firstly, the questionnaire data in this paper was collected only once. However, recent studies on scale development have recommended that questionnaires be collected at least twice. According to the reasonable practice of scale development in previous literature, this paper randomly divided the collected questionnaires into two parts, and carried out EFA and CFA, respectively to ensure the standardization of scale development.

Secondly, the questionnaire in this study was collected in the form of online response, and a small amount of questionnaire data would become invalid due to random filling by the respondents. At the same time, the questionnaire samples collected in this study were only Chinese, and no foreigners were involved.

Thirdly, regarding the scale development of shopping festival atmosphere, this study focused on the efforts of shopping platforms, consumer behavior and feelings. However, this study ignores the influence of different attributes of goods (such as hedonic and practical) on the atmosphere of the shopping festival, as well as the influence on shopping psychology and behavior.

Some scholars have studied "shopping festival atmosphere" (SFA) and found that this factor has a negative impact on consumption intention and behavior. The scale development of SFA in this study provides a theoretical basis for the future research on the consumption behavior of this factor. In the future, the research on SFA can draw on the scale of this study, and analyze the connotation of online or offline SFA. The online shopping festival has entered the era of live e-commerce, and the "shopping festival atmosphere" under this situation has a new connotation, which needs to be further explored. Many scholars have studied the shopping atmosphere of live e-commerce, which also provides a basis for future research.

**6.** Overall, the paper presents a rigorous and comprehensive approach to developing a measurement scale for the atmospheric elements of China's Singles Day Shopping Festivals. The strengths of the study include the multi-method scale development process, the identification of novel atmospheric cues, and the clear discussion of the theoretical and practical implications. To further improve the paper, the authors could strengthen the literature review, provide more details on the methodology, and enhance the discussion by more explicitly connecting the findings to the existing research.

**Response 6:** Finally, thank you very much for your careful review and recognition of our research results. At the same time, we sincerely thank you for your modification suggestions and references.

The above suggestions about " strengthen the literature review ", " more details on the methodology " and "enhance the discussion" have been modified in the corresponding modules and contents in this paper.
